# Emerging epidemics: is the Zanzibar healthcare system ready to detect and respond to mosquito-borne viral diseases?

**DOI:** 10.1186/s12913-021-06867-6

**Published:** 2021-08-24

**Authors:** Fatma Saleh, Jovin Kitau, Flemming Konradsen, Leonard E.G. Mboera, Karin L. Schiøler

**Affiliations:** 1 Department of Parasitology and Entomology, Kilimanjaro Christian Medical University College, Moshi, Tanzania; 2 Department of Allied Health Sciences, School of Health and Medical Sciences, The State University of Zanzibar, Zanzibar, Tanzania; 3 World Health Organization, Country office, Dar es Salaam, Tanzania; 4 Global Health Section, Department of Public Health, University of Copenhagen, Copenhagen, Denmark; 5 SACIDS Foundation for One Health, Sokoine University of Agriculture, Morogoro, Tanzania

**Keywords:** Healthcare system readiness, Mosquito-borne viral disease, Epidemics, Zanzibar

## Abstract

**Background:**

Effective control of emerging mosquito-borne viral diseases such as dengue, chikungunya, and Zika requires, amongst other things, a functional healthcare system, ready and capable of timely detection and prompt response to incipient epidemics. We assessed the readiness of Zanzibar health facilities and districts for early detection and management of mosquito-borne viral disease outbreaks.

**Methods:**

A cross-sectional study involving all 10 District Health Management Teams and 45 randomly selected public and private health facilities in Zanzibar was conducted using a mixed-methods approach including observations, document review, and structured interviews with health facility in-charges and District Health Management Team members.

**Results:**

The readiness of the Zanzibar healthcare system for timely detection, management, and control of dengue and other mosquito-borne viral disease outbreaks was critically low. The majority of health facilities and districts lacked the necessary requirements including standard guidelines, trained staff, real-time data capture, analysis and reporting systems, as well as laboratory diagnostic capacity. In addition, health education programmes for creating public awareness and *Aedes* mosquito surveillance and control activities were non-existent.

**Conclusions:**

The Zanzibar healthcare system has limited readiness for management, and control of mosquito-borne viral diseases. In light of impending epidemics, the critical shortage of skilled human resource, lack of guidelines, lack of effective disease and vector surveillance and control measures as well as lack of laboratory capacity at all levels of health facilities require urgent attention across the Zanzibar archipelago.

## Background

Mosquito*-*borne viral diseases (MBVDs) including dengue, chikungunya, yellow fever, Rift Valley fever and Zika pose important public health threats in the tropical and subtropical regions of the world, in the form of acute, large-scale epidemics including severe morbidity and mortality [1, 2]. In recent decades, dengue has emerged as the most important MBVD causing abrupt and substantial burdens to the healthcare systems of affected countries [3]. In the African region, dengue outbreaks have been recorded since the 1960s [4, 5], with Sub-Saharan Africa accounting for a disproportionally higher global burden of dengue based on model estimates [6]. Over the past 10 years, mainland Tanzania has experienced six outbreaks of dengue, the most recent occurring in 2019, including thousands of reported cases and multiple deaths [7–11]. Chikungunya and Rift Valley fever (RVF) have also been reported in different regions of mainland Tanzania in recent years [12–20], while neighboring Kenya, Uganda and the Democratic Republic of Congo have reported chikungunya and yellow fever [21, 22]. The situation indicates high risk of MBVDs in Zanzibar, accentuated by urbanization, and substantial regional trade and travel [21].

The archipelago of Zanzibar located off the east coast of Tanzania, is yet to officially report any MBVD outbreak. This, even as only a single case of MBVD constitutes an epidemic according to national guidelines [23] and as a 2013 facility-based study confirmed acute dengue virus (DENV) infection by RT-PCR in 9 of 149 (6 %) febrile outpatients presenting at the main referral hospital [24]. In addition, seroprevalence studies among febrile outpatients on Pemba island in 2007, and among healthy blood donors on Unguja island in 2011, reported an anti-DENV IgG prevalence of about 8 % (7/91) and 51 % (253/500), respectively [25, 26]. These findings suggest that circulation of DENVs on the Zanzibar islands is under-recognized, as most likely for other arboviruses. Notably, we recently identified widespread infestation of urban and rural communities on Unguja Island by *Aedes aegypti*, the main vector of dengue, chikungunya, yellow fever and Zika viruses [27, 28].

While available findings [27, 28] suggest an overt risk of epidemic spread of MBVDs throughout all communities of the archipelago, this risk is projected to remain high through 2050 in the coastal regions of Tanzania including Zanzibar due to expanding presence of infected *Aedes aegypti* [29]. As evidenced by the 2005–2006 chikungunya outbreak on La Reunion, large-scale MBVD epidemics can have devastating social and economic consequences to small-island communities such as Zanzibar that are heavily dependent on global tourism [30].

The International Health Regulations (2005) recommend all member states to build resilience against epidemic-prone diseases including strengthening surveillance and preparedness capacity for early detection of outbreaks and by taking timely public health actions for their mitigation and control [3, 31]. The capacity to respond to emerging public health threats is determined by the readiness of the healthcare system in terms of infrastructure, availability of trained staff, standard guidelines, equipment, diagnostic capacity, and supplies needed for provision of the required services [32]. Equally important are governance structures for provision of incentives that motivate service providers to perform [33]. Limited information, if any, is available concerning the capacity of the Zanzibar healthcare system to detect and respond to mosquito-borne viral infections. In this study, we specifically assessed the readiness for early detection and management of dengue and other MBVD outbreaks at district and health facility levels in Zanzibar.

## Materials and methods

### Study design and setting

This cross-sectional study was conducted in the archipelago of Zanzibar (06°00′S, 39°00′E), located about 40 km off the eastern coast of Tanzania mainland. Zanzibar is a semi-autonomous part of the United Republic of Tanzania with its own government named the Revolutionary Government of Zanzibar. According to the 2012 census, Zanzibar has a population of 1,303,569, with an annual growth rate of 3.1 % [34]. About 70 and 30 % of the population reside on the two main islands of Unguja and Pemba, respectively. The majority (70 %) of the population lives in rural areas across the archipelago, with the capital city of Stone Town representing the main urban center [34]. Both Unguja and Pemba Islands were included in the study.

### Zanzibar healthcare system

Zanzibar healthcare system is organized into national, district and health facility levels. In each district there is a District Health Management Team (DHMT) that coordinates all health services in the respective district and serves as a link between health facility and the national level (Ministry of Health) [23]. Public health services in Zanzibar are delivered through a hierarchical structure of health facilities from the community to the national level consisting of primary health care units (PHCU), primary health care centers (PHCC), district hospitals, regional hospital, specialized hospitals (maternity and mental hospitals) and a national referral hospital [35].

The PHCU is the lowest level of the healthcare system and usually provides the first point of patient contact serving an average population of 3000 to 20,000 [36, 37]. PHCUs provide basic primary health care services including curative and preventative services for a defined locality within specified daytime hours [35, 37]. The PHCC denotes a second level primary health care facility providing all services offered by PHCUs, but at a larger scale and operates 24 h a day. The PHCCs serve as a first line of referral from PHCUs serving an average population of 30,000 to 150,000 [36, 37]. The district hospitals provide second-line referral facilities and specialist services.

At the time of the study, Zanzibar had 152 PHCUs, four PHCCs, 2 district hospitals, 2 specialized hospitals (not included in this study), 1 regional, and 1 national referral hospital. There were also 90 private facilities that provide complimentary diagnostic and curative services to a relatively smaller segment of the population [38]. All health facilities in Zanzibar, irrespective of level (except the national referral hospital), provide primary health care services to a specified catchment area. Also, patients may be referred for further services to any of the higher-level facilities depending on availability of the services needed [37].

### Sampling

A stratified random sampling was applied to select public, and private health facilities including PHCUs/dispensaries, PHCCs and hospitals from each of the 10 districts of Zanzibar (Unguja and Pemba). Briefly, for each district, PHCUs were selected using a simple random sampling method, while PHCCs, hospitals and district health offices were purposively sampled due to their small number. A total of 45 health facilities; 30 from Unguja and 15 from Pemba, were included in the assessment. Of these, 5 were hospitals, 4 PHCCs and 36 PHCUs. The PHCCs and PHCUs are henceforth referred to as primary health care facilities. Among the included primary health facilities, 29 were public and 11 were private, whereas for hospitals, four were public and one private. In addition, all 10 DHMT were included. In each health facility or district, the person in-charge or responsible for disease surveillance was interviewed. The details of the methods have been described elsewhere [39].

### Data collection

Data relating to readiness of the districts and health facilities in Zanzibar for detection, management, and control of dengue and other MBVDs were collected during November and December 2017. The data were collected through document reviews, observations, and structured interviews with health facility in-charges and DHMT members using modified World Health Organization generic questionnaires for assessing disease surveillance and response systems [40]. Prior to data collection, the questionnaires were validated and modified by omitting and/or adding relevant assessment indicators during stakeholders meeting. In addition, an open-ended question was included to capture the views of the participants on ways to improve MBVD surveillance and response functions. System readiness was assessed based on the following variables: basic amenities, surveillance, diagnostic capacity, human resource capacity, availability of standard guidelines, as well as prevention and control measures (Table 1).

**Table 1:** Healthcare system readiness indicators assessed for dengue and other MBVDs in Zanzibar (Adapted from WHO [32])

Category	Variable	Description
Basic amenities	Power supply	Routine supply of electricity
	Power generator	For backup power supply
	Communication facilities	Functioning telephone or radio call
	Computer with internet/modem	Functioning computer with access to internet
	Transportation	Routinely available vehicle for emergency transportation
Surveillance	Standard case definitions (SCD)	Presence of SCDs for MBVDs (dengue, chikungunya, yellow fever, zika, RVF)
	Surveillance tools/forms	Adequate supply of forms
	Capacity for data analysis	Visible graphs/charts, tables
	Data reporting system	Existence of electronic/web-based system/database for data reporting
	Information sharing policy/practice	Availability of data sharing practices among sectors, countries
	Surveillance networks	Laboratory networks and coordination for surveillance
	Epidemic preparedness/readiness	Availability of dengue/MBVD contingency plans, rapid response teams, heath education programmes, community involvement in disease surveillance
Diagnostic readiness	Capacity to collect, store and transport blood/serum samples	Availability of sample collection and storage materials
	Availability of functional diagnostic equipment and materials	Rapid diagnostic test kits, molecular diagnostic tools/reagents
Human resource capacity	Availability of trained staff	Health facilities and districts with trained staff on MBVD surveillance, and response (management and control)
Standard guidelines	Availability of national guidelines for dengue/MBVDs	Availability of guidelines for surveillance, sample collection, handling, and transportation, and case management
Prevention and control	Existing prevention and control measures	Availability of vector control guidelines and programme/practices
		Awareness creation programmes/activities

### Data analysis

Data were entered and processed in Microsoft Excel 2010 and descriptive analysis completed in SPSS for Windows version 20.0 (Armonk, NY). Frequency distribution tables were prepared, and proportions calculated based on specific assessment indicators for district and type of health facilities (Tables 2 and 3). Narrative responses from the open-ended question were analyzed thematically.

**Table 2:** Readiness of Zanzibar health care facilities in the detection and response to MBVD outbreaks

Variable/indicator	Primary health care facilities^a^		Secondary health care facilities (Hospitals)	
	Public (***n*** = 29)	Private (***n*** = 11)	Public (***n*** = 4) + Private (***n*** = 1)	Facility Total (***n*** = 45)
	n (%)	n (%)	n (%)	n (%)
Surveillance				
Availability of standard case definitions for MBVDs:				
Dengue, yellow fever	21 (72)	2 (18)	0 (0)	23 (51)
Chikungunya, Zika, Rift Valley Fever (RVF)	0 (0)	0 (0)	0 (0)	0 (0)
Availability of adequate supply of surveillance forms	26 (80)	9 (82)	5 (100)	40 (89)
Capacity for data analysis/visible graphs	3 (10)	0 (0)	0 (0)	3 (7)
Facility with electronic system/database for surveillance data reporting	0 (0)	0 (0)	0 (0)	0 (0)
Standard guidelines				
Availability of national guidelines for MBVD surveillance				
Yellow fever (within national IDSR guidelines)	3 (10)	1 (9)	0 (0)	4 (9)
Dengue, chikungunya, Zika, RVF	0 (0)	0 (0)	0 (0)	0 (0)
Availability of standard case management manuals for MBVDs	0 (0)	0 (0)	0 (0)	0 (0)
Availability of guidelines for sample collection, handling, and transportation	11 (38)	8 (73)	3 (60)	22 (49)
Diagnostic readiness				
Capacity to collect and handle blood/serum samples until transportation	23 (79)	7 (64)	4 (80)	34 (75)
Capacity to transport samples to higher level laboratory	10 (35)	3 (27)	3 (60)	16 (36)
Availability of functional diagnostic equipment and materials				
Rapid diagnostic test kits	0 (0)	0 (0)	0 (0)	0 (0)
Molecular diagnostics	0 (0)	0 (0)	0 (0)	0 (0)
Human resource capacity				
Trained on disease surveillance	18 (62)	4 (36)	2 (40)	22 (49)
Trained on MBVDs surveillance and response (only dengue reported)	2 (7)	0 (0)	0 (0)	2 (4)
MBVD prevention/control measures				
Availability of a contingency plan	0 (0)	0 (0)	0 (0)	0 (0)
Provide routine health education on MBVDs and control measures	0 (0)	0 (0)	0 (0)	0 (0)
Perform routine environmental management practices for *Aedes* mosquito control	0 (0)	0 (0)	0 (0)	0 (0)

^a^PHCUs and PHCCs

**Table 3:** Readiness of districts in the detection and response to MBVD outbreaks in Zanzibar

Variable/indicator	District (*n* = 10)
	n (%)
Surveillance	
Surveillance system in place (routine integrated disease surveillance and response system)	10 (100)
Availability of adequate supply of surveillance tools in the past six months	8 (80)
Electronic system/database for weekly surveillance data reporting	Not available
Regular monitoring and evaluation of surveillance activities	Not conducted
Data sharing policy and practices	Not available
System of intercountry communications during outbreaks	Not available
Laboratory coordination and networks for surveillance	Not available
Standard guidelines	
Availability of national manual for dengue/MBVD surveillance	Not available
Availability of standard case management manual for MBVDs	Not available
Epidemic preparedness/readiness	
Availability of rapid response teams for epidemics	10 (100)
Availability of dengue/MBVD contingency plans	Not available
Community involvement/community-based surveillance	Not practiced
Health education and promotion programme on MBVDs	Not available
Human resource capacity	
Trained on disease surveillance	10 (100)
Trained on MBVD surveillance and response/management (only dengue reported)	5 (50)
MBVD prevention/control measures	
Awareness creation campaigns	Not practiced
Guidelines/manual for *Aedes* mosquito control	Not available
Routines *Aedes* surveillance and control programme/practices	Not available
Available vector control interventions	
Insecticide treated nets, indoor residual spraying, and larviciding	Malaria only

## Results

### Availability of basic amenities at health facilities and districts

**Fig. 1:**
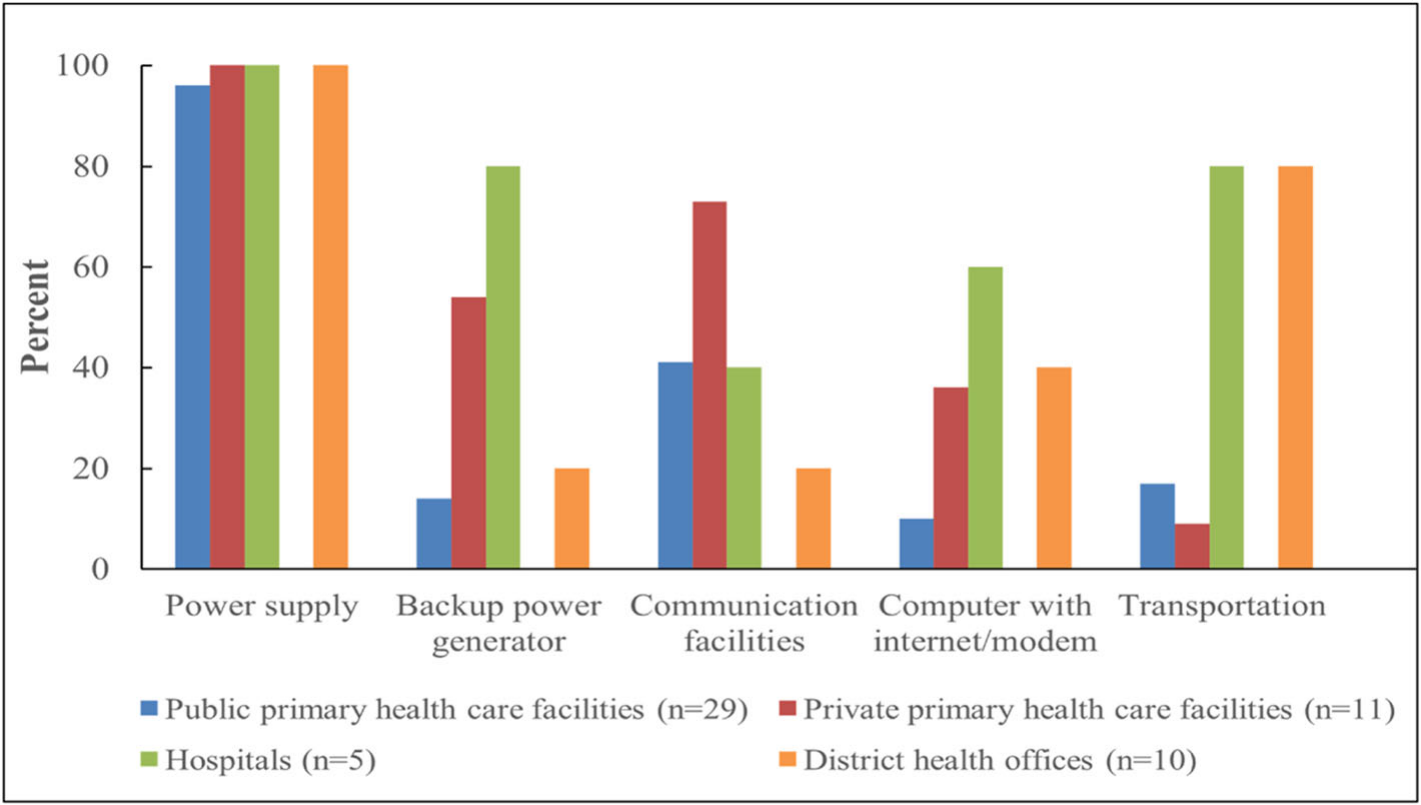
Percentage of health facilities and districts with basic amenities to support mosquito-borne viral disease surveillance and response

All districts (10) and majority of health facilities (98 %) had routine supply of electricity. However, power supply interruptions were reported to be common and only 14/45 (31 %) of the facilities and 20 % (2/10) districts had backup power generators. Less than half of health facilities, 22/45 (49 %) and only 2/10 (20 %) of district offices had communication facilities (telephone service). Internet service (computer with internet/modem) was more available at the hospitals (3/5, 60 %) than public primary (3/29, 10 %), private primary facilities (4/11, 36 %) and districts (4/10, 40 %). Likewise, 80 % (8/10) of districts and hospitals (4/5) had a vehicle/ambulance for emergency transportation, whereas only 5/29 (17 %) and 1/11 (9 %) public and primary health facilities, respectively, had functioning vehicles (Fig. 1). Nevertheless, shortage of fuel was reported to be a constant constraint at all districts and health facilities with vehicles.

### Health facility readiness

#### Mosquito-borne viral disease surveillance

Interviews and document reviews at each health facility revealed that standard case definitions (SCDs) were available for dengue and yellow fever, only. These SCDs were available in the form of printed handbooks in 23 (51 %) health facilities, 21 (72 %) public primary, and 2 (18 %) private primary facilities. None of the hospitals had SCDs for any of the MBVDs (Table 2).

The capacity for data analysis and reporting were observed to be minimal at all levels of health facilities, with only three of 29 (10 %) public primary facilities having visible line graphs in place, mostly for malaria or cholera. None of the private facilities or hospitals conducted their own analyses and none of the facilities had electronic systems/databases for reporting weekly infectious disease data to the district office. Health facility staff reportedly used short message services (SMS) to submit weekly reports, however, lack of airtime voucher and poor telephone networks caused delays or failure to submit.

#### Standard guidelines

During the study period, there was no specific manual for dengue or any MBVD surveillance and response in place. The national Integrated Disease Surveillance and Response (IDSR) guidelines, which mentions dengue, chikungunya and yellow fever among the priority diseases include only a brief synopsis of yellow fever surveillance, with no guidelines for dengue or chikungunya. Moreover, through document review, the national guidelines were found to be outdated, voluminous, and not user-friendly, and were only available in 4/45 (9 %) of the health facilities. Likewise, none of the facilities had a standard case management manual for any MBVD.

#### Diagnostic capacity

Laboratory capacity for mosquito-borne viral infections at all health facility levels were found to be insufficient, with limited availability of skilled staff, equipment, and supplies (including rapid diagnostic test kits). None of the health facilities had the capacity to conduct molecular diagnostic techniques for confirmation of any of the MBVDs. Confirmation of suspected dengue/MBVD cases, required transfer of blood samples to the national laboratory in mainland Tanzania – typically by sea ferry, a journey of approximately four hours. Test results were reported to the Zanzibar Ministry of Health within 48 h or longer.

#### Human resource capacity

A critical scarcity of personnel trained on MBVD surveillance and management was observed at all health facility levels. Only 2 (7 %) of the interviewed public facility in-charges (*n* = 29) were trained on MBVD surveillance and response, mainly during their degree programmes. None of the interviewed staff at private facilities reported to have received training on MBVD surveillance and response.

#### Mosquito-borne viral disease prevention and control measures

Mosquito-borne viral diseases are yet to be recognized as a health priority in Zanzibar as there was no control activity or contingency plan in place. None of the health facilities had routine health education activities or vector control programmes for prevention of dengue and other MBVDs (Table 2).

### District readiness

#### Mosquito-borne viral disease surveillance

The IDSR strategy was introduced in Zanzibar in 2010 involving passive surveillance reliant on routine submission of surveillance forms/reports from health facilities. Majority (8/10, 80 %) of districts reported to have adequate supply of surveillance reporting forms. However, as in the case of health facilities, none of the districts had a web-based computerised system for reporting weekly infectious disease data to the national level, and data analytical capacity was inadequate with only 2 (20 %) districts having visible graphs for data presentation. In addition, there were no regular system for monitoring and evaluation of surveillance activities, information sharing practices, and intercountry communication during outbreaks or for laboratory networking and coordination. Moreover, with the exception of brief guidelines on yellow fever surveillance embedded within the national IDSR guidelines available in 7 (70 %) of the districts, there were no specific manual for MBVD surveillance or standard case management protocols at any of the DHMT offices (Table 3).

#### Epidemic preparedness

Despite the existence of epidemic management committees in 70 % (7/10) of the districts, none had a schedule for regular meetings for revising, updating, or evaluating their epidemic preparedness and response plans or activities. In addition, inadequate budget allocation was common in all districts, affecting epidemic preparedness and response activities including staff training and provision of health education. Furthermore, mosquito-borne viral disease contingency plans were not available, none of the districts had health education and promotion programmes on MBVDs, and community-based surveillance was not practiced (Table 3).

#### Human resource capacity

Staff were inadequately trained on MBVDs at the district level. Only 5 (50 %) of the interviewed district staff reported having received training on MBVD (specifically dengue) surveillance, management, and control either through their degree programmes (2/10, 20 %) or short in-service training offered by the Ministry of Health (3/10, 30 %) (Table 3).

#### MBVD prevention and control

During the study period, none of the districts had *Aedes* mosquito control manual/guidelines or routine surveillance and control programmes in place. Furthermore, none had educational programmes or conducted public awareness campaigns for MBVDs and control measures. The existing vector control interventions organised by Zanzibar Malaria Elimination Programme exclusively targeted malaria vectors, including distribution of long-lasting insecticide treated bed nets (LLINs), periodic indoor residual spraying (IRS) and larviciding in malaria high-risk areas (Table 3).

#### Proposed strategies for enhancing MBVD surveillance and response

The following strategies were proposed by DHMT members when asked for their suggestions on ways to improve MBVD surveillance and response/management: (i) development of a national strategy or programme for MBVD prevention and control, (ii) development of guidelines for MBVD surveillance and response, (iii) case management manuals/guidelines, (iv) capacity building of health personnel at all levels of healthcare system, (v) provision of health education and awareness creation among communities, (vi) sensitization of communities to report suspicious or unusual conditions or events to health facilities, (vii) allocation of adequate budget for outbreak preparedness and response, and (viii) involvement of community health committees and school boards/committees in disease surveillance.

## Discussion

Our study found no evidence of MBVD surveillance or control efforts in Zanzibar, although several MBVDs are listed as reportable priority diseases in the Zanzibar IDSR guidelines [23]. Specifically, we observed a lack of guidelines and training on MBVD surveillance and management as well as a complete absence of laboratory diagnostic capacity at all levels of the healthcare system. Low awareness and knowledge on MBVDs at point of care indicate that frontline health care staff are yet to recognise the diseases as public health threats. As such, they are unlikely to suspect, confirm and/or trigger early warning on MBVD case presentation. Our study informants within the healthcare system attributed the lack of laboratory diagnostics, training and awareness to financial constraints and competing priorities, in line with other reports from the African region [5, 41–43].

To ensure allocation of adequate resources for preparedness and response activities, regular risk assessments of emerging public health threats, including MBVDs, must be part of the national agenda [44]. Specifically, it is recommended that countries develop their own context-specific MBVD contingency plans and national strategy or programme for prevention and control, with an extensive component on awareness creation and community involvement [45, 46]. This aligns with the suggestions offered by the DHMT members in this study, as ways to improve MBVD surveillance and response in Zanzibar.

To achieve enhanced or early outbreak detection, a variety of elements may be combined with basic passive surveillance activities, such as active, sentinel, syndromic and/or community-based surveillance [46–48]. Inclusion of each of these surveillance elements would be difficult to attain in Zanzibar, yet different approaches and combinations should be considered. As an example, Taiwanese health authorities have captured notable numbers of dengue cases through laboratory testing of immediate contacts of confirmed cases identified through passive surveillance, in addition to school-based reporting of febrile students and fever screening of airport arrivals [49, 50]. Reportedly, airport screening alone captured almost 45 % of imported cases during the 4-year surveillance period from 2007 to 2010 [50].

The use of hospitals as sentinels of early disease activity is gaining increased attention, given recent reports of hospital-acquired dengue outbreaks occurring well in advance of notable community transmission [51, 52]. We consider routine monitoring of febrile episodes among patients and staff a feasible approach to sentinel surveillance in Zanzibar and suggest that both private and public hospitals be engaged to ensure widest possible coverage.

Intersectoral collaboration between the healthcare system, private pharmacies and local schools for syndromic surveillance of fever cases is a recommended approach by the WHO [45, 46, 53] for early detection of incipient epidemics across a wide population spectrum. To fully operate in Zanzibar, this approach would require improved reporting and feedback mechanisms within the existing IDSR system before extension to other actors.

Community-based surveillance (CBS) aimed at “community participation in detecting, reporting, responding to and monitoring health events in the community” is an important approach for early detection of epidemic-prone diseases and unusual public health events occurring in communities [54, 55]. CBS is useful for rumor reporting and detection of health events in communities that do not seek care at health facilities including hard-to-reach areas which would otherwise been missed [55]. Thus, integration of CBS within the existing IDSR system should be considered and guidelines for its operationalization established.

The success of any of the above surveillance approaches will depend on the availability of timely and reliable laboratory diagnosis of mosquito-borne viral infections. Notably, this study did not include a detailed stocktaking at public and private facilities in terms of existing human resources, laboratory capacity and training for undertaking laboratory diagnosis of MBVDs. However, to the best of our knowledge, there are no established operations in Zanzibar for routine diagnosis of suspected MBVDs. As such, there is a clear need to assess whether diagnostic capacity should be established at point of need or whether centralized diagnostic facilities would be more appropriate to the Zanzibar context.

Importantly, development of spatio-temporal prediction models that utilize climatic, socioecological, vector and epidemiological data may find increasing use for early warning [29, 56]. Integration of such models within the existing District Health Information System (DHIS2) would provide an important platform for monitoring and forecasting of epidemic risks of MBVDs in Zanzibar and is subject to current research activities across mainland Tanzania and Zanzibar [57].

For effective MBVD prevention and epidemic response, disease surveillance efforts should be congruent with vector surveillance and control. However, health authorities in Zanzibar, like other sub-Saharan African countries, focus almost exclusively on malaria, leaving MBVD largely neglected. In Zanzibar, vector control interventions against malaria currently include the use of LLINs, periodic IRS and larviciding in rice paddies and other water bodies. These efforts have contributed to a dramatic reduction of the malaria prevalence from 40 % in 2005 to < 1 % in 2020 [58–60]. Unfortunately, these interventions are ineffective against the diurnal-biting and container-breeding *Aedes aegypti.* To address the ‘double burden’ of vector-borne diseases, public health authorities must consider policy reforms and mobilization of resources to implement interventions targeting the ecologically diverse vectors and pathogens in an integrated manner, as recommended by the WHO [61].

It is worth emphasizing that vector control is the mainstay of MBVD prevention and control. Unless efforts to control vector populations are undertaken, the risk of epidemics will remain high. Several vector control interventions including biological control, chemical control, and environmental management, targeting either adult or larvae stages, have been tested for reducing vector populations in different settings [62]. As adult mosquitoes are developing resistance towards the commonly applied chemical insecticides as observed in malaria vectors in Zanzibar [58], effective *Aedes* control can be achieved largely through integrated vector management approaches involving environmental management targeting larval source reduction [3]. In Cuba, Toledo et al. [63] reported decreased household risk behaviors, reduction of environmental risk factors as well as reduction of entomological indices following a community-based participatory vector control intervention involving behavior change communication and environmental management practices. Similarly, in India, a community-based vector control intervention using a community-based environmental management approach including provision of water container covers, clean-up campaigns, and dissemination of dengue information through schoolchildren yielded significant gains in the reduction of entomological indices and increased dengue knowledge among community members [64]. It would be prudent for Zanzibar to follow these or similar approaches while developing its own context-specific and targeted *Aedes* surveillance and control program. For any mosquito-borne disease control intervention to be successful and sustainable, it should be collectively organized, accepted by the community and implemented with their full participation [65, 66]. Top-down approaches have proven challenging in mosquito control endeavors, thus efforts to mobilize and involve relevant sectors and potential actors at all levels of society are crucial [66–69].

## Conclusions

Our study provides important information for strengthening the readiness of the Zanzibar healthcare system for detection and management of MBVDs. The system is generally weak and has low readiness for timely detection and response to MBVD epidemics. Identified weaknesses include, an inadequate surveillance system, low staff capacity, lack of standard guidelines for MBVD prevention, management and control, and lack of laboratory diagnostic capacity at all levels of the healthcare system. Competing priorities and financial constraints offer some explanation to the observed situation and underscore the need for reassessment of priorities and institutionalized approach to in-service and regular training of staff at all levels of the healthcare system. Likewise, strengthening both disease and vector surveillance and control, and enhancing laboratory diagnostic capacity are paramount and urgent. This includes development and communication of standard guidelines. To enhance disease surveillance, we recommend that the existing IDSR system is supplemented by one or more of the suggested surveillance approaches. In addition, social mobilization, and involvement of communities in surveillance and control is crucial and having contingency plans in place would provide a framework for public health authorities to take the right and timely actions to minimize morbidity and mortality during future MBVD outbreaks.

## Data Availability

All data generated and analysed during this study are included in this article. Data access provided on request to the corresponding author (FS).

## References

[1] Weetman D, Kamgang B, Badolo A, Moyes CL, Shearer FM, Coulibaly M, et al. Aedes mosquitoes and Aedes -bornearboviruses in Africa: current and future threats. Int J Environ Res Public Heal. 2018;15. 10.3390/ijerph15020220 PMC5858289 29382107

[2] Licciardi S, Loire E, Cardinale E, Gislard M, Dubois E, Cêtre-Sossah C. In vitro shared transcriptomic responses of Aedesaegypti to arboviral infections: Example of dengue and Rift Valley fever viruses. Parasites Vectors. 2020;13. 10.1186/s13071-020-04253-5 PMC7404916 32758286

[3] World Health Organization (2012). Global Strategy for Dengue Prevention and Control 2012–2020.

[4] Brady OJ, Gething PW, Bhatt S, Messina JP, Brownstein JS, Hoen AG (2012). Refining the global spatial limits of dengue virus transmission by evidence-based consensus. PLoS Negl Trop Dis.

[5] Cattarino L, Rodriguez-Barraquer I, Imai N, Cummings DAT, Ferguson NM. Mapping global variation in dengue transmissionintensity. Sci Transl Med. 2020;12:eaax4144. 10.1126/scitranslmed.aax4144 31996463

[6] Cattarino L, Rodriguez-Barraquer I, Imai N, Cummings DAT, Ferguson NM. Mapping global variation in dengue transmission intensity. Sci Transl Med. 2020;12. 10.1126/scitranslmed.aax4144 31996463

[7] Mboera LEG, Mweya CN, Rumisha SF, Tungu PK, Stanley G, Makange MR (2016). The risk of dengue virus transmission in Dar es Salaam, Tanzania during an epidemic period of 2014. PLoS Negl Trop Dis.

[8] Ward T, Samuel M, Maoz D, Runge-Ranzinger S, Boyce R, Toledo J (2017). Dengue data and surveillance in Tanzania: a systematic literature review. Trop Med Int Heal.

[9] PRO/EAFR. Dengue - Tanzania (Dar es Salaam). Archive Number: 20180320.5697356. 2018. https://www.promedmail.org/. Accessed 22 Mar 2018.

[10] Okada K, Morita R, Egawa K, Hirai Y, Kaida A, Shirano M (2019). Dengue virus type 1 infection in traveler returning from Tanzania to Japan, 2019. Emerg Infect Dis.

[11] ProMED-EAFR. Dengue – Tanzania (04): increase in cases. Archive Number: 20190730.6594542. 2019. http://www.promedmail.org/eafr. Accessed 24 Dec 2019.

[12] Hertz JT, Lyaruu LJ, Ooi EE, Mosha FW, Crump JA (2016). Distribution of Aedes mosquitoes in the Kilimanjaro Region of northern Tanzania. Pathog Glob Health.

[13] Hertz J, Munishi O, Ooi E, Howe S, Lim W, Chow A (2012). Chikungunya and dengue fever among hospitalized febrile patients in northern Tanzania. Am J Trop Med Hyg.

[14] Kajeguka DC, Kaaya RD, Mwakalinga S, Ndossi R, Ndaro A, Chilongola JO, et al. Prevalence of dengue and chikungunyavirus infections in north-eastern Tanzania: a cross sectional study among participants presenting with malaria-like symptoms. BMC Infect Dis. 2016;16. 10.1186/s12879-016-1511-5 PMC4845349 27112553

[15] Crump JA, Morrissey AB, Nicholson WL, Massung RF, Stoddard RA, Galloway RL (2013). Etiology of severe non-malaria febrile illness in northern Tanzania: a prospective cohort study. PLoS Negl Trop Dis.

[16] Weller N, Clowes P, Dobler G, Saathoff E, Kroidl I, Ntinginya NE (2014). Seroprevalence of Alphavirus antibodies in a cross- sectional study in southwestern Tanzania suggests endemic circulation of chikungunya. PLoS Negl Trop Dis.

[17] Chipwaza B, Mugasa JP, Selemani M, Amuri M, Mosha F, Ngatunga SD (2014). Dengue and chikungunya fever among viral diseases in outpatient febrile children in Kilosa district hospital, Tanzania. PLoS Negl Trop Dis.

[18] Kinimi E, Shayo MJ, Patrick BN, Angwenyi SO, Kasanga CJ, Weyer J (2018). Evidence of chikungunya virus infection among febrile patients seeking healthcare in selected districts of Tanzania. Infect Ecol Epidemiol.

[19] Sindato C, Karimuribo ED, Pfeiffer DU, Mboera LEG, Kivaria F, Dautu G (2014). Spatial and Temporal Pattern of Rift Valley Fever Outbreaks in Tanzania; 1930 to 2007. PLoS One.

[20] Budodo RM, Horumpende PG, Mkumbaye SI, Mmbaga BT, Mwakapuja RS, Chilongola JO (2020). Serological evidence of exposure to rift valley, dengue and chikungunya viruses among agropastoral communities in manyara and morogoro regions in Tanzania: A community survey. PLoS Negl Trop Dis.

[21] World Health Organization. Yellow fever in Africa and the Americas, 2016. Wkly Epidemiol Rec. 2017;32:437–52.

[22] World Health Organization. Chikungunya – Mombasa, Kenya. Disease outbreak news, 27 February 2018. WHO. 2018. www.who.int/csr/don/27-february-2018-chikungunya-kenya/en/. Accessed 1 Mar 2019.

[23] Ministry of Health Zanzibar. Zanzibar guidelines for integrated disease surveillance and response (IDSR). Ministry of Health Zanzibar; 2010.

[24] Ali MA, James OC, Mohamed AA, Joachim A, Mubi M, Omodior O. Etiologic agents of fever of unknown origin among patients attending Mnazi Mmoja hospital, Zanzibar. J Community Health. 2020;45:1073-80. 10.1007/s10900-020-00832-w 32399732

[25] Vairo F, Nicastri E, Meschi S, Schepisi MS, Paglia MG, Bevilacqua N (2012). Seroprevalence of dengue infection: A cross-sectional survey in mainland Tanzania and on Pemba Island, Zanzibar. Int J Infect Dis.

[26] Vairo F, Nicastri E, Yussuf SM, Cannas A, Meschi S, Mahmoud MAA (2014). IgG against dengue virus in healthy blood donors, Zanzibar, Tanzania. Emerg Infect Dis.

[27] Saleh F, Kitau J, Konradsen F, Alifrangis M, Lin C-H, Juma S (2018). Habitat characteristics for immature stages of Aedes aegypti in Zanzibar City, Tanzania. J Am Mosq Control Assoc.

[28] Saleh F, Kitau J, Konradsen F, Kampango A, Abassi R, Schiøler KL (2020). Epidemic risk of arboviral diseases: Determining the habitats, spatial-temporal distribution, and abundance of immature Aedes aegypti in the Urban and Rural areas of Zanzibar, Tanzania. PLoS Negl Trop Dis.

[29] Mweya CN, Kimera SI, Stanley G, Misinzo G, Mboera EG (2016). Climate change influences potential distribution of infected Aedes aegypti co- occurrence with dengue epidemics risk areas in Tanzania. PLoS One..

[30] Soumahoro M-K, Boelle P-Y, Gaü Zere B-A, Atsou K, Pelat C, Lambert B (2011). The Chikungunya Epidemic on La Ré union Island in 2005–2006: A Cost-of-Illness Study. PLoS Negl Trop Dis.

[31] World Health Organization. International Health Regulations (2005): Areas of work for implementation. World Health Organization; 2007.

[32] World Health Organization. Service Availability and Readiness Assessment (SARA). An annual monitoring system forservice delivery: Reference Manual. Geneva: World Health Organization; 2015.

[33] The World Bank. Tanzania Service Delivery Indicators: Education | Health. Washington DC: The World Bank; 2012.

[34] The United Republic of Tanzania. 2012 Population and Housing Census. Population Distribution by Administrative Areas. The United Republic of Tanzania; 2013.

[35] Revolutionary Government of Zanzibar. Ministry of Health Zanzibar. Zanzibar Health Sector Strategic Plan III 2013/14-2018/19. Ministry of Health Zanzibar; 2013.

[36] Human Resource for Health Division. Ministry of Health Zanzibar. Minimum Staffing Requirements. Ministry of Health Zanzibar; 2014.

[37] Ministry of Health Zanzibar. Review of the essential health care package in Zanzibar. Ministry of Health Zanzibar; 2007.

[38] Ministry of Health Zanzibar. Zanzibar annual health bulletin 2018. Ministry of Health Zanzibar; 2019.

[39] Saleh F, Kitau J, Konradsen F, Mboera LEG, Schiøler KL. Assessment of the core and support functions of the integrateddisease surveillance and response system in Zanzibar, Tanzania. BMC Public Health. 2021;21. 10.1186/s12889-021-10758-0 PMC8052932 33865347

[40] World Health Organization (2001). Protocol for the Assessment of National Communicable Disease Surveillance and Response Systems: Guidelines for Assessment Teams.

[41] Mboera LEG, Sindato C, Mremi IR, George J, Ngolongolo R, Rumisha SF, et al. Socio-ecological systems analysis ofprevention and control of Dengue in two Districts of Dar es Salaam City, Tanzania. Infect Ecol Epidemiol. 2021 (in press).

[42] Masiira B, Nakiire L, Kihembo C, Katushabe E, Natseri N, Nabukenya I, et al. Evaluation of integrated disease surveillanceand response (IDSR) core and support functions after the revitalisation of IDSR in Uganda from 2012 to 2016. BMC Public Health. 2019;19. 10.1186/s12889-018-6336-2 PMC6327465 30626358

[43] Fall IS, Rajatonirina S, Yahaya AA, Zabulon Y, Nsubuga P, Nanyunja M, et al. Integrated disease surveillance and response(IDSR) strategy: current status, challenges and perspectives for the future in Africa. BMJ Glob Heal. 2019;4:e001427. 10.1136/bmjgh-2019-001427 PMC6615866 31354972

[44] WHO (2017). Joint external evaluation of IHR core capacities of the United Republic of Tanzania - Zanzibar.

[45] Runge-Ranzinger S, Kroeger A, Olliaro P, McCall PJ, Sánchez Tejeda G, Lloyd LS (2016). Dengue Contingency Planning: From Research to Policy and Practice. PLoS Negl Trop Dis.

[46] World Health Organization (2016). Technical handbook for dengue surveillance, dengue outbreak prediction/detection and outbreak response (“model contingency plan”).

[47] Runge-Ranzinger S, McCall PJ, Kroeger A, Horstick O (2014). Dengue disease surveillance: An updated systematic literature review. Trop Med Int Heal.

[48] Badurdeen S, Valladares DB, Farrar J, Gozzer E, Kroeger A, Kuswara N, et al. Sharing experiences: Towards an evidencebased model of dengue surveillance and outbreak response in Latin America and Asia. BMC Public Health. 2013;13. 10.1186/1471-2458-13-607 PMC3697990 23800243

[49] Lin CH, Schiøler KL, Jepsen MR, Ho CK, Li SH, Konradsen F (2012). Dengue outbreaks in high-income area, Kaohsiung city, Taiwan, 2003–2009. Emerg Infect Dis.

[50] Kuan MM, Chang FY. Airport sentinel surveillance and entry quarantine for dengue infections following a fever screeningprogram in Taiwan. BMC Infect Dis. 2012;12. 10.1186/1471-2334-12-182 PMC3462143 22867003

[51] Almeida-Nunes J, Marcilio I, Oliveira MS, Gonçalves EMN, Batista MV, Mendrone A (2016). Hospital-acquired vector-transmitted dengue fever: an overlooked problem?. Infect Control Hosp Epidemiol.

[52] Ehelepola NDB, Wijesinghe WMCM. An analysis of a dengue outbreak at a large hospital and epidemiological evidence fornosocomial dengue. J Trop Med. 2018;2018:9579086. 10.1155/2018/9579086 PMC6038582 30046313

[53] World Health Organization. Communicable disease surveillance and response systems: Guide to monitoring and evaluating.World Health Organization; 2006.

[54] World Health Organization Regional Office for Africa (2014). Integrated disease surveillance and response in the African region. A guide for establishing community based surveillance: Disease surveillance and response programme area. Disease prevention and control cluster.

[55] Technical Contributors to the June 2018 WHO (2019). A definition for community-based surveillance and a way forward: Results of the WHO global technical meeting, France, 26 to 28 June 2018. Eurosurveillance.

[56] Hussain-Alkhateeb L, Kroeger A, Olliaro P, Rocklöv J, Sewe MO, Tejeda G (2018). Early warning and response system (EWARS) for dengue outbreaks: Recent advancements towards widespread applications in critical settings. PLoS One.

[57] Predicting the next epidemic: DHIS2-based risk modeling | Danida Reseach Portal. http://drp.dfcentre.com/project/predictingthe-next-epidemic-dhis2-based-risk-modeling/. Accessed 19 Mar 2021.

[58] Haji KA, Khatib BO, Smith S, Ali AS, Devine GJ, Coetzee M, et al. Challenges for malaria elimination in Zanzibar:pyrethroid resistance in malaria vectors and poor performance of long-lasting insecticide nets. Parasit Vectors. 2013;6. 10.1186/1756-3305-6-82 PMC3639098 23537463

[59] Björkman A, Shakely D, Ali AS, Morris U, Mkali H, Abbas AK, et al. From high to low malaria transmission in Zanzibar —challenges and opportunities to achieve elimination. BMC Med. 2019;17. 10.1186/s12916-018-1243-z PMC6341737 30665398

[60] Zanzibar Malaria Elimination Program (ZAMEP). Annual Report 2019–2020, Ministry of Health Zanzibar; 2020.

[61] WHO (2012). Handbook for integrated vector management.

[62] WHO (2003). Guidelines for dengue surveillance and mosquito control.

[63] Toledo ME, Vanlerberghe V, Baly A, Ceballos E, Valdes L, Searret M (2007). Towards active community participation in dengue vector control: results from action research in Santiago de Cuba, Cuba. Trans R Soc Trop Med Hyg.

[64] Arunachalam N, Tyagi BK, Samuel M, Manavalan R, Tewari SC, Ashokkumar V, et al. Community-based control of Aedesaegypti by adoption of eco-health methods in Chennai City, India. Pathog Glob Health. 2012;106:8,488-496. 10.1179/2047773212Y.0000000056 PMC3541894 23318241

[65] Andersson N, Nava-aguilera E, Arosteguí J, Morales-perez A, Suazo-laguna H, Legorreta-soberanis J, et al. Evidence basedcommunity mobilization for dengue prevention in Nicaragua and Mexico (Camino Verde, the Green Way): cluster randomized controlled trial. Br Med J. 2015;351:h3267. 10.1136/bmj.h3267 PMC4495677 26156323

[66] Parks WJ, Lloyd LS, Nathan MB, Hosein E, Odugleh A, Clark GG, et al. International experiences in social mobilization andcommunication for dengue prevention and control. Dengue Bull. 2004;28(suppl.):1-7.

[67] World Health Organization (2017). Global Vector Control Response 2017–2030.

[68] Herdiana H, Sari JFK, Whittaker M (2018). Intersectoral collaboration for the prevention and control of vector borne diseases to support the implementation of a global strategy: A systematic review. PLoS One..

[69] Parks W, Lloyd L (2004). Planning social mobilization and communication for dengue fever prevention and control: A step-by-step guide.

